# Effect of ranibizumab on retinopathy of prematurity: A meta-analysis

**DOI:** 10.3389/fphar.2022.897869

**Published:** 2022-08-22

**Authors:** Zhibin Wang, Zhaobo Zhang, Yue Wang, Yu Di

**Affiliations:** ^1^ Department of Ophthalmology, Shengjing Hospital of China Medical University, Shenyang China; ^2^ Department of Cardiology, First Hospital of China Medical University, Shenyang, China

**Keywords:** laser therapy, meta-analysis, ranibizumab, randomized controlled trials, retinopathy of prematurity, review

## Abstract

The primary objective of this study was to systematically evaluate the clinical efficacy of intravitreal ranibizumab injection in the treatment for retinopathy of prematurity (ROP) in infants. The MEDLINE (PubMed), Embase, China Biology Medicine disc, Cochrane Library, Web of Science, WanFang Data, CNKI, and CQVIP databases were searched to collect randomized controlled trials (RCTs) comparing the efficacy of ranibizumab with laser treatment in ROP. The retrieval time was from 2007, on which ranibizumab was approved until 12 January 2022. Data were extracted based on predetermined inclusion and exclusion criteria. Two investigators employed QUADAS-2 to independently assess the quality of all eligible original studies. Following quality evaluation, we also performed a meta-analysis using STATA v 15.1 and RevMan v 5.4 and funnel plots were used to detect publication bias. A total of five RCTs were included in the meta-analysis. In this study, the regression rate of retinal neovascularization was used as the index of therapeutic effectiveness. According to the results, the retinal neovascularization regression rate of the intravitreal ranibizumab injection group was statistically higher than that of the laser therapy group [risk ratio (RR) = 1.26, 95% confidence interval (CI): 1.18–1.35]; however, the incidence of adverse events, including recurrence and complications, was not different between them (RR = 0.73, 95%CI: 0.19–2.80). Therefore, intravitreal ranibizumab injection may be more clinically effective than laser therapy in the treatment for ROP. The safety and efficacy of ranibizumab in the long-term treatment for ROP needs further investigation.

**Systematic Review Registration**: https://www.crd.york.ac.uk/prospero/, CRD42022296387

## 1 Introduction

Retinopathy of prematurity (ROP) affects some preterm infants with low birth weight and exposure to high oxygen supplementation, which may lead to blindness in severe cases. Pathological progression of ROP begins at the immature stage of retinal vascular and neuronal development in preterm infants (stage I), followed by tissue ischemia leading to hypoxia-induced neovascularization (stage II) (Xu et al., 2018; Wang et al., 2019). Mild ROP resolves spontaneously with few sequelae, but severe ROP can lead to retinal detachment, severe visual impairment, and blindness. With constant developments in perinatal medicine the survival rate of preterm and low birth weight infants are improving. However, the incidence of ROP remains high, with approximately 28,300–45,600 infants being diagnosed annually with irreversible visual impairment due to ROP worldwide (Blencowe et al., 2013). Currently, cryotherapy, fusion laser photocoagulation, and vitreous injections are mostly used to reduce peripheral retinal neovascularization (RNV) (Marlow et al., 2021). Laser or cryotherapy is commonly used in children with lesions up until stage III, while vitrectomy or scleral buckling is often required following stage IV and onwards. Although laser therapy is standard, it can lead to extensive and permanent destruction of the retina and blood vessels, leading to the loss of peripheral vision (Rishi and Rishi, 2019). Vascular endothelial growth factor (VEGF) is of great significance in the occurrence and development of ROP. Usually, the VEGF concentration in the vitreous is high in children with ROP, which provides a theoretical basis for clinical anti-VEGF therapy (Sankar et al., 2018). The anti-VEGF monoclonal antibody, ranibizumab, can inhibit the expression of VEGF. This may control the intraocular neovascularization and subsequently achieve the goal of treatment of ROP (Mitchell et al., 2011; Aranda et al., 2019). This study aims to provide a basis for guiding clinical decision-making by exploring the effective rate and incidence of adverse events in the treatment of ROP, by comparing ranibizumab with laser therapy through a meta-analysis.

## 2 Materials and methods

### 2.1 Search strategy

Two researchers searched MEDLINE (PubMed), Embase, Chinese Biomedical Literature Database (CBM), The Cochrane Library, Web of Science, WanFang Data, CNKI, and VIP database for relative literature. Randomized controlled trials (RCTS) were conducted to compare ranibizumab with laser therapy in the treatment of ROP. The search time is set to build from database construction until to 12-01-2022. Subsequently, each reviewer manually re-evaluated whether the delivered literature fit the theme of the meta-analysis used in this study. English search terms included: ROP (MeSH terms), ROP (All Fields), Prematurity Retinopathies (All Fields), Prematurity Retinopathy (All Fields), Retrolental Fibroplasia (All Fields), Fibroplasia Retrolental (All Fields), Fibroplasias Retrolental (All Fields), and Retrolental Fibroplasias (All Fields). The above search terms are connected by OR, followed by AND with Ranibizumab (MeSH terms), RhuFab V2 (All Fields), V2 RhuFab (All Fields), and Lucentis (All Fields). Two researchers then conducted a review of all preliminary studies eligible for inclusion in the study to determine whether other relevant studies were included in the study.

### 2.2 Study selection and eligibility criteria

The inclusion criteria comprised of the following conditions: 1) The types of studies were a RCT; 2) The subjects were premature infants (gestational age <37 weeks) diagnosed with ROP by binocular indirect ophthalmoscope and retinal camera (RetCam); 3) The experimental group was given intravitreal injection of ranibizumab, and the control group was given laser treatment; 4) Outcome indicators were the number of effective treatment cases (including neovascularization subsided and bleeding decreased, additional lesions were alleviated, blood circulation was restored to the vascular occlusion area, and no intraocular infection and adverse events occurred) and the number of adverse events (including recurrence, high myopia, amblyopia, glaucoma, and other complications); 5) Published in English or Chinese. Exclusion criteria comprised of the following criteria: 1) The full text of the study was not available; 2) Literature on inconsistent interventions or outcome measures; 3) Non-Chinese or non-English literature; 4) Duplicate reports and studies without original data; 5) Literature without outcome indicators or where outcome indicators were not available.

### 2.3 Quality assessment

Cochrane literature quality evaluation tool was used to evaluate the quality of the included RCTS. The evaluation included whether random assignment was used, whether the assignment was hidden, whether blinding was used, whether the results were complete, whether the results were reported selectively, and whether there were other sources of bias. There are “low risk,” “high risk,” and “unclear” judgments for every project. Two researchers independently evaluated the quality of the five included studies.

### 2.4 Statistical analysis

RevMan v 5.3 and STATA v 16.0 software were used for statistical analysis. Dichotomous variables (response rate and incidence of adverse events) were analyzed by risk ratio (RR) and 95% confidence interval (CI). *p* < 0.05 indicated statistically significant differences. The heterogeneity was determined by the chi-square test and I^2^ to make a quantitative judgment. If no statistical heterogeneity among the results (I^2^ <50%), the fixed effect model was used. If there were statistical heterogeneity (I^2^ ≥50%), the random effects model for meta-analysis were used to find the source of the heterogeneity. For obvious clinical heterogeneity, we used subgroup analysis and sensitivity analysis, or only a descriptive analysis. Egger’s method was used to test the publication bias. STATA 16.0 was used for meta regression to find the cause of heterogeneity.

## 3 Results

### 3.1 Characteristics of eligible literatures

**FIGURE 1:**
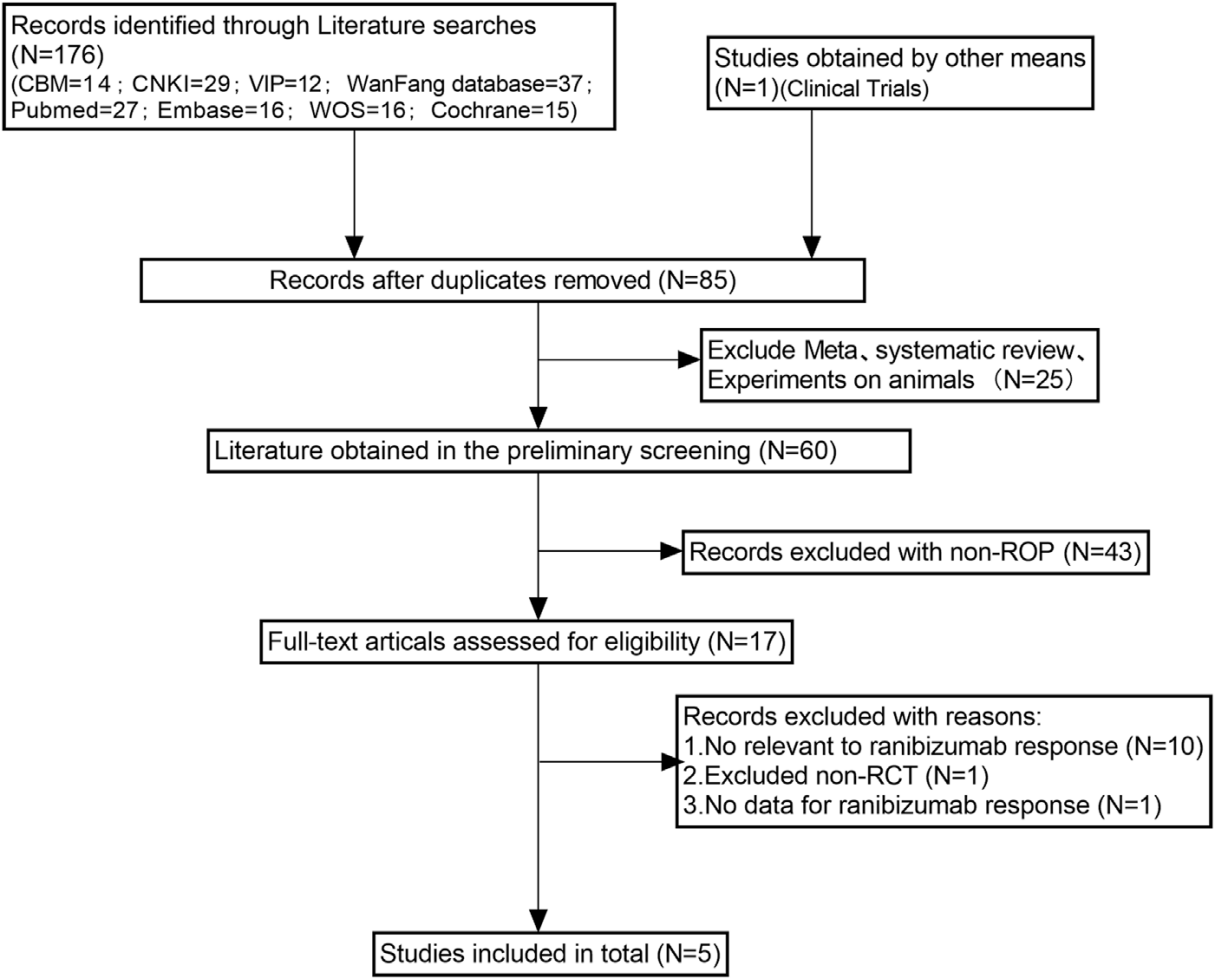
Literature screening process and results.

The literature screening process and results are shown in Figure 1. We removed 92 duplicates from the original 177 articles retrieved from the databases. Subsequently, we excluded 68 unrelated articles and a variety of non-RCTs. Then, in order to further evaluate whether the remaining 17 studies met the conditions of our study, we obtained the full text of these 17 studies, and the results showed that 10 studies were not related to ranibizumab, one study had no data on ranibizumab response, and one study was a non-RCT study. Finally, we conducted quantitative analysis on five articles (Chen et al., 2018; Shi and Chen, 2018; Sun and Zhang, 2018; Stahl et al., 2019; Xin, 2021) that met the inclusion requirements.

### 3.2 The basic characteristics of the literature included in the study and the evaluation results of bias risk

**FIGURE 2:**
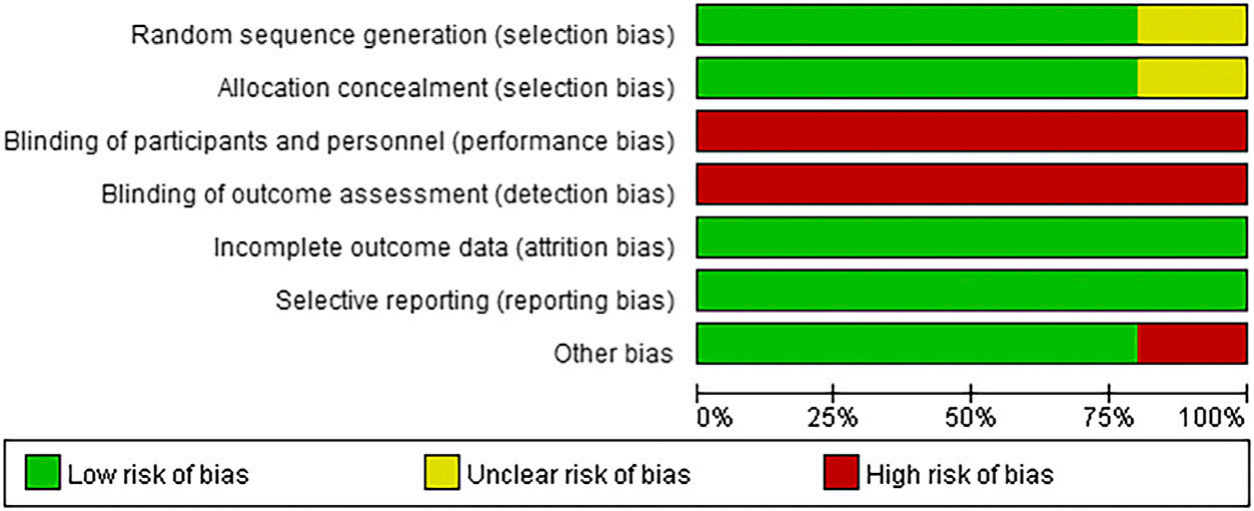
Risk assessment of bias in all included studies:risk of bias graph.

**FIGURE 3:**
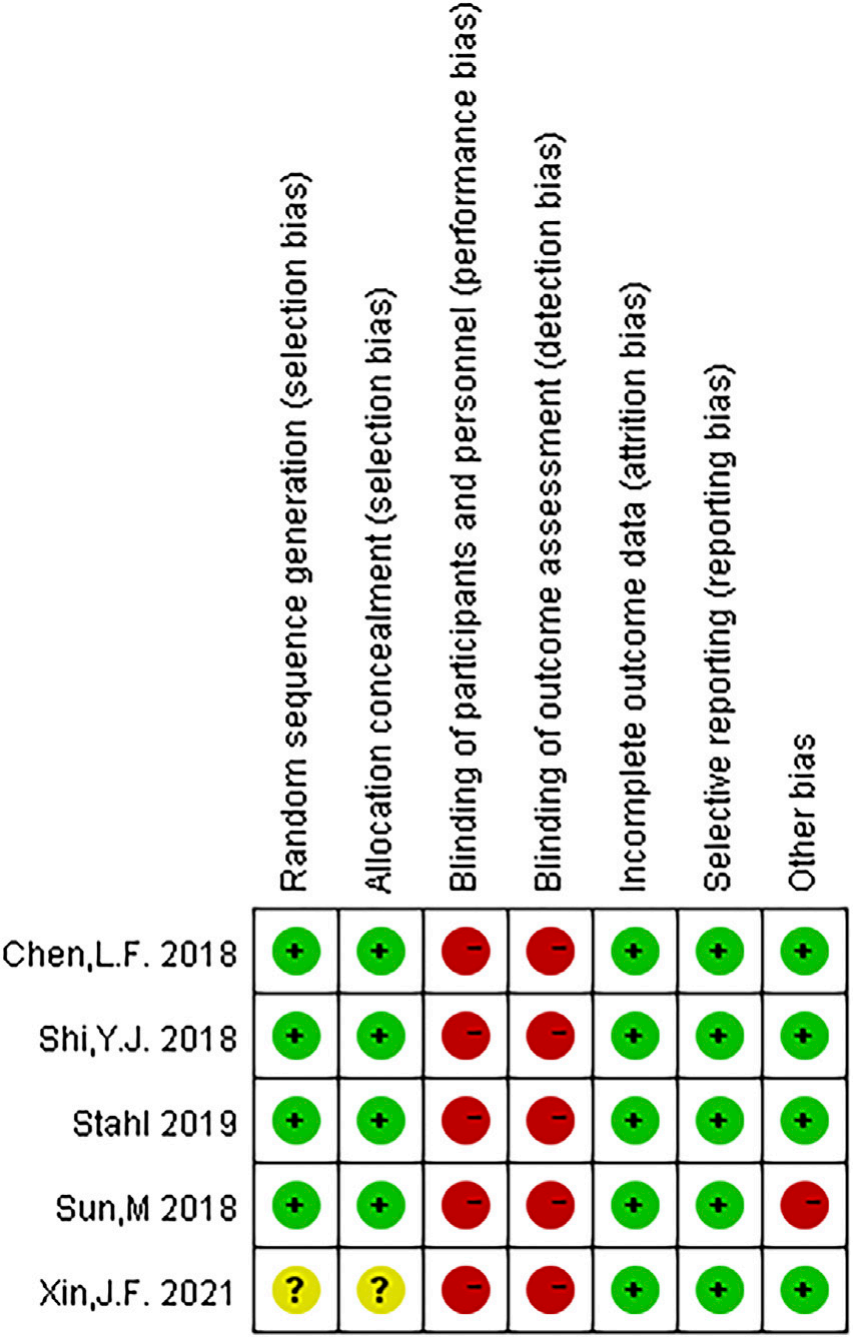
Risk assessment of bias in all included studies:risk of bias summary.

Table 1 summarizes the relevant characteristics of the final five studies. The results of the quality evaluation chart made by the investigator with RevMan v 5.4 are shown in Figures 2, 3. All five eligible studies obtained moderate scores in the Quality assessment of Cochrane literature quality evaluation tool, indicating that the included RCTs have an overall medium risk of bias.

**TABLE 1:** Basic characteristics of each study.

Included studies	Sample size (T/C, eye)	Correct gestational age (T/C, week)	Weight (T/C, kilogram)	Interventions		Follow-up time (month)	Outcome indicators
				T	C		
Xin (2021)	80/80	34.31 ± 1.31/34.18 ± 1.35	2.85 ± 0.43/2.84 ± 0.32	Ranibizumab	Laser therapy	6	①②
Shi,Y. J. (2018)	104/102	28.9 ± 1.30/28.93 ± 1.33	1.40 ± 0.20/1.39 ± 0.21	Ranibizumab	Laser therapy	1	①②
Chen L. F. (2018)	80/80	37.40 ± 1.75/36.93 ± 1.84	1.45 ± 0.20/1.42 ± 0.21	Ranibizumab	Laser therapy	1	①②
Sun.M. (2018)	40/40	30.1 ± 3.3/30.7 ± 3.5	1.53 ± 0.51/1.51 ± 0.40	Ranibizumab	Laser therapy	1	①②
Stahl (2019)	292/136	—	—	Ranibizumab	Laser therapy	6	①②

PS:T: Ranibizumab group; C: Laser group; ①Effective; ②Adverse events (include recurrency, complications).

### 3.3 Meta-analysis

#### 3.3.1 Effective rate

**FIGURE 4:**
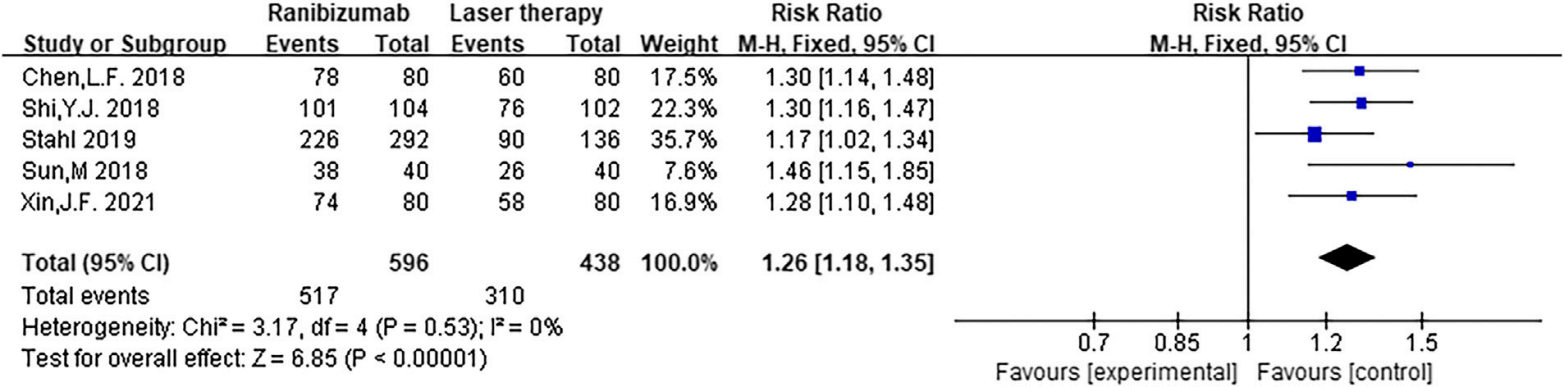
Forest plot: ranibizumab injection of retinopathy of prematurity (ROP) efficacy compared to laser treatment.

A total of five RCTs were included in the meta-analysis of this study (Chen et al., 2018; Shi and Chen, 2018; Sun and Zhang, 2018; Stahl et al., 2019; Xin, 2021). The effective rate tested for heterogeneity in all included studies delivered I^2^ = 0% while the Q test showed *p* = 0.53, which indicated no heterogeneity among the literatures selected for this study (that is, heterogeneity does not have statistical significance). The fixed effect is selected to combine the effect size. The subsequent analysis indicates that the effective rate of ranibizumab injection was higher than that of laser therapy, and the difference was statistically significant [RR = 1.26, 95%CI (1.18, 1.35), z = 6.85, *p* < 0.00001] (Figure 4), which indicates that the efficacy of ranibizumab in the treatment of ROP was significantly better than treating with laser therapy alone, and the effective rate of intravitreal ranibizumab injection was 1.26 times that of laser treatment.

#### 3.3.2 Adverse event rate

**FIGURE 5:**
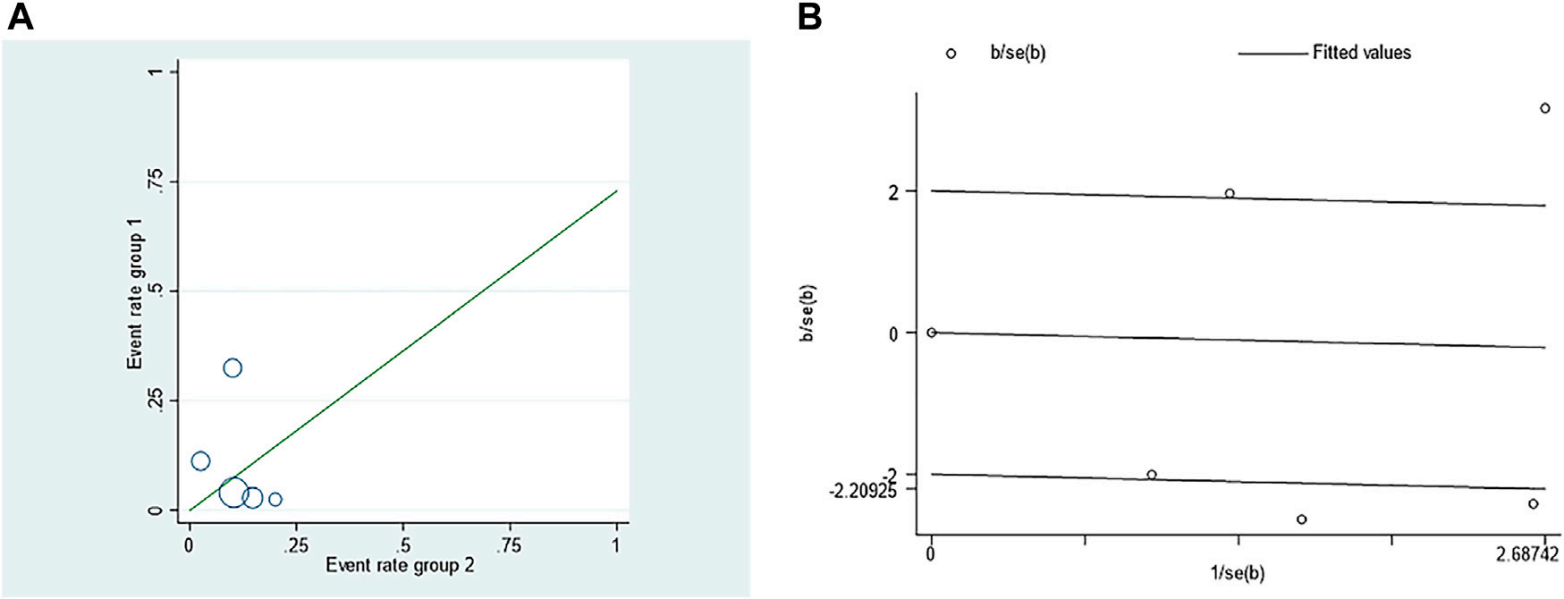
Investigation of the heterogeneity in the incidence of adverse events in retinopathy of prematurity (ROP) treated with ranibizumab and laser therapy: **(A)** Labbe Plot **(B)** Galbraith Radial Plot.

**FIGURE 6:**
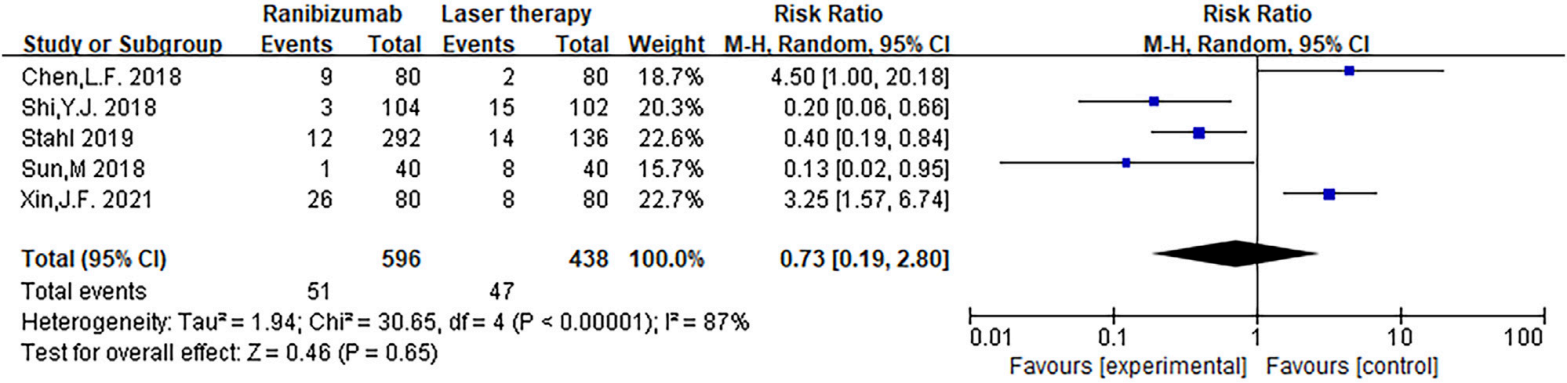
Forest plot: ranibizumab injection of retinopathy of prematurity (ROP) incidence of adverse events compared to laser treatment.

The adverse event rate tested for heterogeneity in all included studies delivered I^2^ = 86.9% while the *Q* test showed *p* <0.00001, which suggested that the heterogeneity among the literature selected in this study was statistically significant. The adverse event rates were then further inspected at Labbe Plot and Galbraith Radial Plot (Figure 5). By graphical analysis, we concluded that there is a moderate heterogeneity among the literature in this study, and the effect size can be combined with random effect. The final result for the analysis of adverse event rates delivered RR = 0.73 [0.19, 2.80], meaning the number of adverse events in the intervention group were a total of 73% compared with those in the control group, but were not statistically significant (z = 0.46, *p* = 0.65) (Figure 6). This suggested that although ranibizumab could reduce the adverse events of ROP, there was no statistically significant difference in the incidence of adverse events between ranibizumab and the control measure (laser treatment).

#### 3.3.3 Meta-regression

**FIGURE 7:**
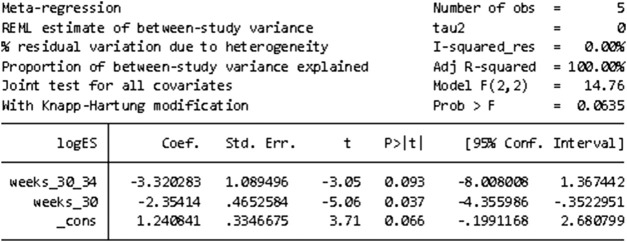
Results of meta-regression to find the cause of heterogeneity in the incidence of adverse events.

STATA16.0 was used for meta-regression analysis of the causes of heterogeneity in the incidence of adverse events. According to the number of weeks of corrected gestational age, the study was divided into three groups: 30–34 weeks, less than 30 weeks, and 34–36 weeks, respectively. Meta-regression was conducted using group (number of corrected gestational age) variables as covariate, and the results suggested that the number of corrected gestational age was the source of heterogeneity. However, due to the small number of RCTS included, subgroup studies cannot be carried out. Figure 7 shows the results of meta-regression.

### 3.4 Sensitivity analysis and publication bias

**FIGURE 8:**
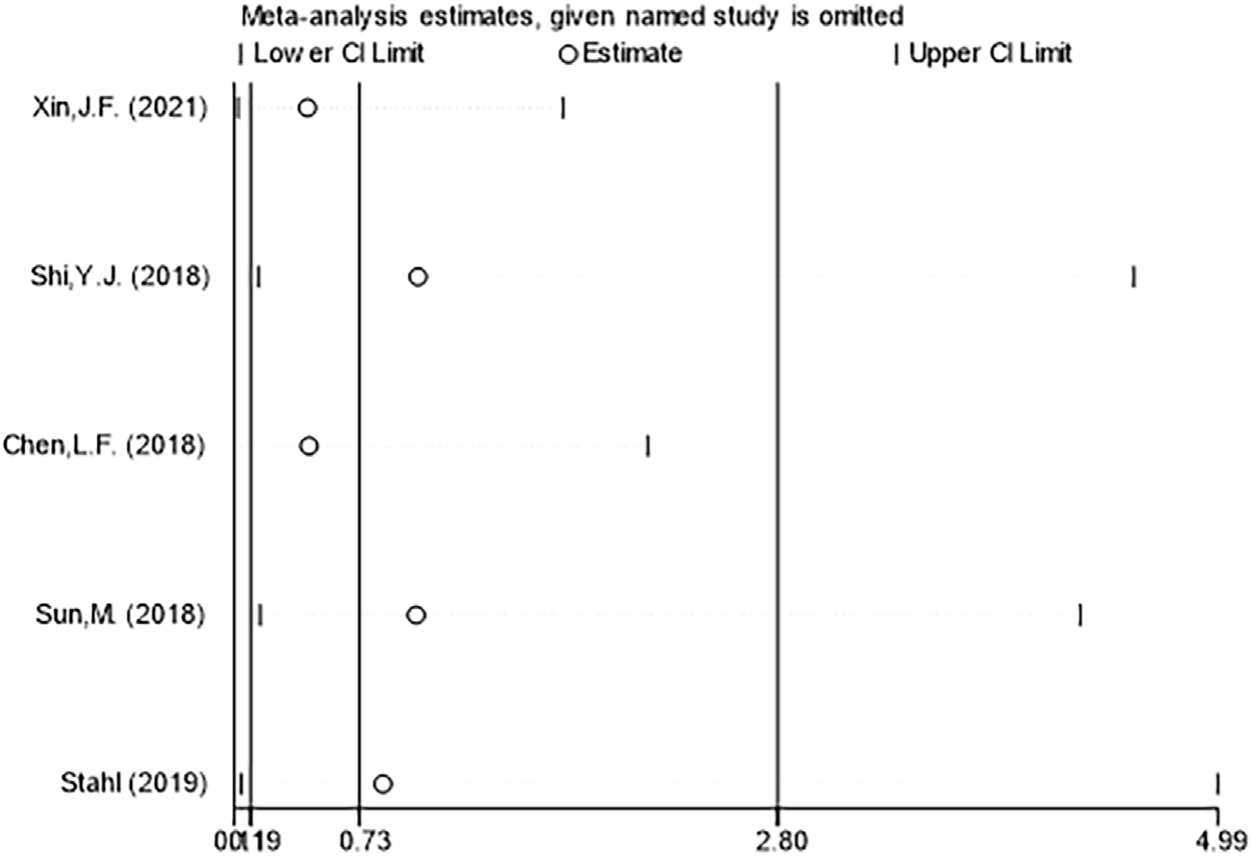
Sensitivity analysis of the incidence of adverse events between ranibizumab and laser therapy for retinopathy of prematurity (ROP).

**FIGURE 9:**
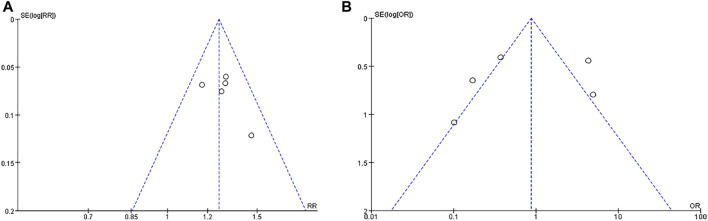
Funnel plot indicating publication bias for treatment effectiveness **(A)** and adverse events rate **(B)**.

Sensitivity analysis was used to find out the heterogeneous causes, and sensitivity analysis was conducted on the five manuscripts used in this study. It was concluded that there was no study with a large impact on heterogeneity. Figure 8 clearly illustrates that deletion of any study did not result in a significant change in results, so the sensitivity analysis did not examine the source of heterogeneity. A funnel plot was used to investigate whether there was publication bias in the five manuscripts used in this study. To test the bias of treatment effectiveness we obtained funnel plot symmetry (Egger’s test showed *p* = 0.364) and to test the bias of adverse events rate (Egger’s test showed *p* = 0.652) we also obtained funnel plot symmetry (Figure 9). Therefore, we concluded that there was no publication bias, which indicated that the conclusion of this study was accurate and reliable.

## 4 Discussion

ROP is a secondary complication of prematurity generally due to oxygen supplementation in NICU, in preterm infants with severe pulmonary, brain or heart problems. It is more common in premature and low weight infants, which can lead to amblyopia, cataracts, retinal detachment, etc. This usually have serious effects on the quality of life of premature infants, such as impacts on their language, movement, and social adaptability, while bringing heavy burden to the family and society (Dogra et al., 2017). Because of its serious consequences, ROP has become the focus of ophthalmological research worldwide. The pathogenesis is actually clear, while the risk factor are not univocally identified. In the past, condensation and photocoagulation were usually used in the treatment of ROP, which mainly damage non-vascular structures in the retina of children, so as to reduce the oxygen consumption of retina metabolism, and then achieve the effect of reducing the neovascularization growth factor induced by ischemia and hypoxia, and finally achieve the purpose of inhibiting the development of RNV and controlling the disease of children (Ge et al., 2021). However, laser is often accompanied by a series of complications such as undertreatment, overtreatment (retinal burn, retinal hiatus, exudative retinal detachment), vitreous hemorrhage, corneal burn, as well as the high technical skill required of ophthalmologists, which limits its wide application in clinical practice. In recent years, a large number of studies have reported the efficacy and safety analysis of anti-VEGF (ranibizumab) treatment compared with laser therapy for ROP, with contrasting results (Hosseini et al., 2009; Mintz-Hittner et al., 2011; Lepore et al., 2014; Karkhaneh et al., 2016; Zhang et al., 2017; Stahl et al., 2019).

Intravitreal injection of anti-VEGF drugs has become the preferred treatment for ROP (Chiang, 2018). Ranibizumab is a recombinant humanized anti-VEGF antibody fragment (Fab), which is an inhibitor of angiogenesis (Itatani et al., 2018). It is also known that VEGF is an important factor in the development of neonatal retinopathy (Uemura et al., 2021), and the mechanism of action of ranibizumab is clear it blocks the VEGFR signaling by binding to VEGFA, which means intravitreal ranibizumab injection can inhibit the expression of VEGF, reduce the generation of RNV, and recast newly generated new blood vessels (Bhandari et al., 2020), so as to play an important role in the treatment of ROP and ensure the function of the retina, which has good clinical effect (Lee and Shirley, 2021; Woo et al., 2021). The use of anti-VEGF drugs in the treatment of tissue damage is light, technically easy and quick to administer, and can safely treat critically ill patients presenting with refractive stroma turbidity (Sankar et al., 2018). In certain cases, anti-VEGF treatment even has more of the curative effect required for laser photocoagulation treatment (Stahl et al., 2019). Barry et al., 2021). At the turn of the century, it has been approved for the treatment of ocular neovascular diseases drugs. Furthermore, it can quickly penetrate the retina layer, has a small molecular weight, excludes segments of Fe, and has the advantage of diminished immune response (Dhoot and Kaiser, 2012; Jiang and Mieler, 2017). While anti-VEGF drugs have a distinct advantage in some cases (e.g., zone I disease or aggressive ROP), there are also disadvantages to this treatment, for example, after this treatment, the recurrence rate is still not low, which means anti-VEGF drugs do not effectively reduce the recurrence rate of the disease (Sankar et al., 2018), and often incomplete retinal vascularization, requiring vigilance and prolonged follow-up consultations (VanderVeen et al., 2017). According to the Cochrane review report, although the risk of early retinal dysstructure was reduced from 47.9% to 28.1%, and peripheral retinal ablation in early childhood was associated with a 13.6% reduction in the risk of visual impairment (Sankar et al., 2018). The treatment effect was remarkable, the recurrence rate was low, and the clinical application experience was extensive, which is still the current gold standard for ROP treatment. However, laser operation is more invasive than anti-VEGF intravitreal injections, and the general condition of patients may deteriorate after treatment (Anderson et al., 2014). Photocoagulation scarring affects peripheral visual field, and the high myopia rate increases after treatment (Anderson et al., 2014; VanderVeen et al., 2017; Yang et al., 2021).

This meta-analysis was conducted to evaluate the effective rate and incidence of adverse events in the treatment of ROP with ranibizumab and laser treatment. The results showed that the recovery rate in the ranibizumab group was higher than that in the laser therapy group, and the difference was statistically significant (*p* < 0.05), which suggests that the use of ranibizumab is clinically more effective in the treatment of ROP. There was no statistical difference in the incidence of adverse events between the two groups (*p* > 0.05), and the results may be biased due to the influence of follow-up time and follow-up indicators such as visual field assessment. Due to the small number of RCTs included, more high-quality clinical studies are needed for further verification. In addition, late recurrence of ROP represents a challenge during the follow-up phase and regular follow-ups should be emphasized. Otherwise, ROP recurrence will still occur in children with strabismus, amblyopia, retinal detachment and other complications. According to previous studies (Hartnett, 2017), follow-up of children with retinal vascularization or corrected gestational age of 45 weeks, no threshold lesions, retinal vessels have developed to zone 3 can be terminated if one of the above indications is met.

Shortcomings and prospects of this study: 1) The number of studies on some outcome indicators was small, and the outcome indicators were scattered; 2) The included indices were the number of cases and the number of researchers, and the results were inconsistent with the given parameters, requiring separate analysis; 3) The efficacy of different doses of ranibizumab may be different, and some studies have shown that 0.2 mg ranibizumab is more effective in the treatment of ROP (Stahl et al., 2019); 4) The treatment of ROP varies among different zones. Some studies have shown that anti-VEGF is more advantageous in the treatment of ROP zone I, but laser is better in the treatment of ROP zone II (Kuo et al., 2015). Because of the small number of studies and scattered outcome indicators, subgroup analysis was not feasible; 5) The study lacked comparisons with more established treatments such as vitreoretinal surgery; 6) Long-term effects on neurodevelopmental and functional ocular outcomes after treatment with ranibizumab or laser therapy were not included in this research.

## Data Availability

The raw data supporting the conclusion of this article will be made available by the authors, without undue reservation.

## Abbreviations

RNV, retinal neovascularization; ROP, retinopathy of prematurity.

## References

[B1] Anderson M. F. Ramasamy B. Lythgoe D. T. Clark D. (2014). Choroidal thickness in regressed retinopathy of prematurity. Eye (Lond) 28 (12), 1461–1468. 10.1038/eye.2014.207 25277303 PMC4268454

[B2] Aranda J. V. Qu J. Valencia G. B. Beharry K. D. (2019). Pharmacologic interventions for the prevention and treatment of retinopathy of prematurity. Semin. Perinatol. 43 (6), 360–366. 10.1053/j.semperi.2019.05.009 31153620

[B3] Barry G. P. Yu Y. Ying G. S. Tomlinson L. A. Lajoie J. Fisher M. (2021). Retinal detachment after treatment of retinopathy of prematurity with laser versus intravitreal anti-vascular endothelial growth factor. Ophthalmology 128 (8), 1188–1196. 10.1016/j.ophtha.2020.12.028 33387554 PMC8819483

[B4] Bhandari S. Nguyen V. Fraser-Bell S. Mehta H. Viola F. Baudin F. (2020). Ranibizumab or aflibercept for diabetic macular edema: Comparison of 1-year outcomes from the fight retinal blindness! Registry. Ophthalmology 127 (5), 608–615. 10.1016/j.ophtha.2019.11.018 31932092

[B5] Blencowe H. Lawn J. E. Vazquez T. Fielder A. Gilbert C. (2013). Preterm-associated visual impairment and estimates of retinopathy of prematurity at regional and global levels for 2010. Pediatr. Res. 74 (1), 35–49. 10.1038/pr.2013.205 24366462 PMC3873709

[B6] Chen L. F. Gao H. S. Wang G. Q. , Effects of intravitreal injection of ranibizumab on serum levels of vascular endothelial growth factor, insulin-like growth factor and glutamate in children with retinopathy of prematurity. Maternal Child Health Care China, 2018. 33(02): p. 349–352.

[B7] Chiang M. F. (2018). How does the standard of care evolve? Anti-vascular endothelial growth factor Agents in retinopathy of prematurity treatment as an example. Ophthalmology 125 (10), 1485–1487. 10.1016/j.ophtha.2018.04.018 30243329 PMC12286704

[B8] Dhoot D. S. Kaiser P. K. (2012). Ranibizumab for age-related macular degeneration. Expert Opin. Biol. Ther. 12 (3), 371–381. 10.1517/14712598.2012.660523 22309606

[B9] Dogra M. R. Katoch D. Dogra M. (2017). An update on retinopathy of prematurity (ROP). Indian J. Pediatr. 84 (12), 930–936. 10.1007/s12098-017-2404-3 28674824

[B10] Ge G. Zhang Y. Zhang M. (2021). Pregnancy-induced hypertension and retinopathy of prematurity: A meta-analysis. Acta Ophthalmol. 99 (8), e1263–e1273. 10.1111/aos.14827 33611839

[B11] Hartnett M. E. (2017). Advances in understanding and management of retinopathy of prematurity. Surv. Ophthalmol. 62 (3), 257–276. 10.1016/j.survophthal.2016.12.004 28012875 PMC5401801

[B12] Hosseini H. Khalili M. R. Nowroozizadeh S. (2009). Intravitreal injection of bevacizumab (Avastin) for treatment of stage 3 retinopathy of prematurity in zone I or posterior zone II. Retina 29 (4), 562. 10.1097/IAE.0b013e31819a98a9 19262431

[B13] Itatani Y. Kawada K. Yamamoto T. Sakai Y. (2018). Resistance to anti-angiogenic therapy in cancer-alterations to anti-VEGF pathway. Int. J. Mol. Sci. 19 (4), 1232. 10.3390/ijms19041232 PMC5979390 29670046

[B14] Jiang Y. Mieler W. F. (2017). Update on the use of anti-VEGF intravitreal therapies for retinal vein occlusions. Asia. Pac. J. Ophthalmol. 6 (6), 546–553. 10.22608/APO.2017459 29204993

[B15] Karkhaneh R. Khodabande A. Riazi-Eafahani M. Roohipoor R. Ghassemi F. Imani M. (2016). Efficacy of intravitreal bevacizumab for zone-II retinopathy of prematurity. Acta Ophthalmol. 94 (6), e417–20. 10.1111/aos.13008 27009449

[B16] Kuo H. K. Sun I. T. Chung M. Y. Chen Y. H. (2015). Refractive error in patients with retinopathy of prematurity after laser photocoagulation or bevacizumab monotherapy. Ophthalmologica. 234 (4), 211–217. 10.1159/000439182 26393895

[B17] Lee A. Shirley M. (2021). Ranibizumab: A review in retinopathy of prematurity. Paediatr. Drugs 23 (1), 111–117. 10.1007/s40272-020-00433-z 33447937

[B18] Lepore D. Quinn G. E. Molle F. Baldascino A. Orazi L. Sammartino M. (2014). Intravitreal bevacizumab versus laser treatment in type 1 retinopathy of prematurity: Report on fluorescein angiographic findings. Ophthalmology 121 (11), 2212–2219. 10.1016/j.ophtha.2014.05.015 25001158

[B19] Marlow N. Stahl A. Lepore D. Fielder A. Reynolds J. D. Zhu Q. (2021). 2-year outcomes of ranibizumab versus laser therapy for the treatment of very low birthweight infants with retinopathy of prematurity (RAINBOW extension study): Prospective follow-up of an open label, randomised controlled trial. Lancet. Child. Adolesc. Health 5 (10), 698–707. 10.1016/S2352-4642(21)00195-4 34391532

[B20] Mintz-Hittner H. A. Kennedy K. A. Chuang A. Z. (2011). Efficacy of intravitreal bevacizumab for stage 3+ retinopathy of prematurity. N. Engl. J. Med. 364 (7), 603–615. 10.1056/NEJMoa1007374 21323540 PMC3119530

[B21] Mitchell P. Bandello F. Schmidt-Erfurth U. Lang G. E. Massin P. Schlingemann R. O. (2011). The RESTORE study: Ranibizumab monotherapy or combined with laser versus laser monotherapy for diabetic macular edema. Ophthalmology 118 (4), 615–625. 10.1016/j.ophtha.2011.01.031 21459215

[B22] Rishi E. Rishi P. (2019). Macular hole following successful stage 4B/stage 5 retinopathy of prematurity surgery. Indian J. Ophthalmol. 67 (6), 971–973. 10.4103/ijo.IJO_719_18 31124537 PMC6552571

[B23] Sankar M. J. Sankar J. Chandra P. Bhat V. Srinivasan R. (2018). Anti-vascular endothelial growth factor (VEGF) drugs for treatment of retinopathy of prematurity. Cochrane Database Syst. Rev. 1 (1), CD009734. 10.1002/14651858.CD009734.pub2 29308602 PMC6491066

[B24] Shi Y. J. Chen L. M. (2018). Effect of ranibizumab injection on serum VEGF and IGF-1 levels in children with retinopathy of prematurity. Inn. Mong. Med. J. 50 (10), 1235–1236.

[B25] Stahl A. Lepore D. Fielder A. Fleck B. Reynolds J. D. Chiang M. F. (2019). Ranibizumab versus laser therapy for the treatment of very low birthweight infants with retinopathy of prematurity (RAINBOW): An open-label randomised controlled trial. Lancet 394 (10208), 1551–1559. 10.1016/S0140-6736(19)31344-3 31522845 PMC12316478

[B27] Sun M. Zhang Y. P. , Clinical effect of vitreous injection of ranibizumab in the treatment of retinopathy of prematurity. Contemp. Med., 2018. 24(19): p. 135–137.

[B28] Uemura A. Fruttiger M. D'Amore P. A. De Falco S. Joussen A. M. Sennlaub F. (2021). VEGFR1 signaling in retinal angiogenesis and microinflammation. Prog. Retin. Eye Res. 84 (9), 100954. 10.1016/j.preteyeres.2021.100954 33640465 PMC8385046

[B29] VanderVeen D. K. Melia M. Yang M. B. Hutchinson A. K. Wilson L. B. Lambert S. R. (2017). Anti-vascular endothelial growth factor therapy for primary treatment of type 1 retinopathy of prematurity: A report by the American academy of ophthalmology. Ophthalmology 124 (5), 619–633. 10.1016/j.ophtha.2016.12.025 28341474

[B30] Wang Z. Liu C. H. Huang S. Chen J. (2019). Wnt Signaling in vascular eye diseases. Prog. Retin. Eye Res. 70 (5), 110–133. 10.1016/j.preteyeres.2018.11.008 30513356 PMC6545170

[B31] Woo S. J. Veith M. Hamouz J. Ernest J. Zalewski D. Studnicka J. (2021). Efficacy and safety of a proposed ranibizumab biosimilar product vs a reference ranibizumab product for patients with neovascular age-related macular degeneration: A randomized alinical trial. JAMA Ophthalmol. 139 (1), 68–76. 10.1001/jamaophthalmol.2020.5053 33211076 PMC7677876

[B32] Xin J. F. (2021). Effect of intravitreal injection of ranibizumab on clinical efficacy and retinal functional development in children with retinopathy of prematurity. Prog. Mod. Biomed. 20 (01), 131–134.

[B33] Xu Y. Lu X. Hu Y. Yang B. Tsui C. K. Yu S. (2018). Melatonin attenuated retinal neovascularization and neuroglial dysfunction by inhibition of HIF-1α-VEGF pathway in oxygen-induced retinopathy mice. J. Pineal Res. 64 (4), e12473. 10.1111/jpi.12473 29411894

[B34] Yang X. Y. Cai Y. T. Li Y. , Interpretation of “clinical guidelines for anti-VEGF therapy in retinopathy of prematurity” by Japanese ophthalmology society. Chin. J. Exp. Ophthalmol., 2021. 39(11): p. 1003–1009.

[B35] Zhang G. Yang M. Zeng J. Vakros G. Su K. Chen M. (2017). Comparison of intravitreal injection of ranibizumab versus laser therapy for zone ii treatment-requiring retinopathy of prematurity. Retina 37 (4), 710–717. 10.1097/IAE.0000000000001241 27529839 PMC5388026

